# Combining Treatment of Acute Malnutrition With Integrated Community Case Management: A Cluster‐Randomised Controlled Trial (SETiPlus)

**DOI:** 10.1111/mcn.70203

**Published:** 2026-05-24

**Authors:** Gael Cronin, Mohamed Jelle, Shukri Ali, Yuusuf Siciid, Yayha Abdillahi, Meftuh Omer, Adan Yusuf Mahdi, Saed Abdi Ibrahim, Lilly Scholfield, Emily Keane, Khalid Ali Ahmed, Andrew Seal

**Affiliations:** ^1^ UCL Institute for Global Health, Institute of Child Health London UK; ^2^ Save the Children International Hargeisa, Somaliland Africa; ^3^ Save the Children London UK; ^4^ Ministry of Health Development Hargeisa, Somaliland Africa

## Abstract

Achieving good treatment coverage and outcomes for acutely malnourished children is challenging in resource limited and remote settings. Inclusion of treatment within integrated community case management (iCCM) programmes has shown promise, but evidence is limited. We conducted a cluster randomised controlled trial in rural villages within the districts of Hargeisa, Gabiley, and Faraweyne in Somaliland. We compared treatment coverage and outcomes between 18 villages implementing iCCM, where cases of malnutrition (mid‐upper arm circumference (MUAC) < 12.5 cm and/or oedema) were referred to MCH clinics, and 19 villages implementing iCCM +, where malnutrition treatment was provided by family health workers (FHW) at village level. Coverage was measured in a closed household cohort at baseline and after 6 months of intervention. Treatment outcomes were determined in an open cohort of cases that were identified by the iCCM FHW and followed up on each month. The prevalence of acute malnutrition diagnosed using MUAC was four‐fold lower compared to that diagnosed using weight‐for‐height z‐scores (WHZ) (3% vs. 13%). This larger than expected difference resulted in the study being underpowered. Treatment coverage was 27% and 23% in the control and intervention arms at baseline and increased sharply in the intervention arm with an adjusted difference of 20 percentage points. However, the improvement was not statistically significant (adjusted OR 2.55 (95% CI 0.4, 18.8) *p* = 0.344). Cases were more likely to start treatment in the intervention arm (difference 71.9% (95% CI 58.1, 85.7) *p* < 0.001), but relapse and non‐response were more frequent (*p* = 0.041 & *p* = 0.004). Over diagnosis of acute malnutrition by FHW was high, with 67.4% and 77.4% of cases being misdiagnosed in the control and intervention arm, respectively. While the integration of malnutrition treatment with iCCM shows potential for improving treatment coverage in some contexts, careful consideration should be given to the level of training and supervision required.

**Trial Registration:**

ISRCTN31437934. Registered 3/10/2023.

## Summary

- Using community health workers to provide treatment for acute child malnutrition is one approach to improving access to care in remote areas with limited health services.
- We conducted a trial in rural Somaliland to determine if treatment coverage and outcomes could be improved by including malnutrition management within the integrated community case management (iCCM) package of services.
- Cases identified in intervention (iCCM +) villages were more likely to start treatment. However, diagnostic accuracy was low, resulting in over‐treatment.
- Due to the unexpectedly low number of cases with a MUAC < 12.5 cm, the study was underpowered to determine whether treatment coverage improved.

**Abbreviations**
BSFP: Blanket Supplementary Feeding Programme
CI: confidence interval
FHW: Family Health Worker (local name for Community Health Worker)
GAM: global acute malnutrition
HAZ: height/length‐for‐age z‐score
iCCM: integrated community case management
iCCM + : integrated community case management with addition of malnutrition treatment
MUAC: mid‐upper arm circumference
OTP: outpatient therapeutic programme
RUSF: ready‐to‐use supplementary food
RUTF: ready‐to‐use therapeutic food
SAM: severe acute malnutrition
SC: stabilisation centre
SFP: supplementary feeding programme
TSFP: targeted supplementary feeding programme
WHO: World Health Organisation
WHZ: weight‐for‐length/height z‐score

## 1 Introduction

Ending hunger and malnutrition is of critical importance for global health, as stated in the Sustainable Development Goal number 2. However, the steady decline in the global prevalence of child undernutrition seen over previous decades has been interrupted during the past 5 years, with a worrying increase in stunting. Today, it is estimated that, globally, over 1 in 5 children under the age of 5 is stunted (23.2% (150.2 million), and 6.6% of all children under the age of 5 are wasted (42.8 million) (UNICEF. WHO. World_Bank. 2025). It is also important to note that the drastic reduction in international assistance seen in the last 2 years is a key factor which is impacting efforts to improve malnutrition prevention and treatment (Haseeb and Ali 2026).

In this context, building evidence on effective and cost‐efficient acute malnutrition treatment protocols and delivery mechanisms is essential. In 2020, UN agencies identified treatment coverage as unacceptably low and called for treatment to be ‘…made more readily available and accessible to all who need it regardless of the context’ (WHO 2020). Efforts have been made to increase treatment coverage by simplifying and varying different elements in the protocol such as the diagnostic criteria, and use and dose of different feeding products, resulting in a number of diverse treatment approaches (Bita 2025).

However, while there is evidence on the effectiveness (Bailey et al. 2020; Cazes et al. 2022) and cost‐effectiveness (Cichon et al. 2023. 2025a) of various simplified protocols, the extent to which these protocol changes have resulted in improvements in coverage is mixed and still emerging (Charle‐Cuéllar et al. 2023; Charle‐Cuellar et al. 2021). Recommendations for the delivery of acute malnutrition treatment for children have evolved over time, progressively moving away from inpatient settings to home‐based management and smaller, more widely dispersed distribution sites for therapeutic food. The recognition and scale‐up of the Community‐based Management of Acute Malnutrition (CMAM) approach in 2007, in which treatment moved from inpatient wards to out‐patient clinics or nutrition sites, resulted in a pivotal change in the treatment of malnutrition, substantially increasing children's potential access to care (Collins et al. 2006; WHO. WFP. UNSCN. UNICEF. 2007).

CMAM includes outpatient programmes where caregivers can bring their child home and give them ready‐to‐use therapeutic foods (RUTF), eliminating the need for a healthcare worker's constant presence. These programmes are, however, still centred around health centres or distribution points, which can be hours away from the patients' home. A lack of physical proximity and the opportunity cost of attending are two of the barriers to access that have been regularly described in studies on malnutrition treatment. A 2019 analysis found that less than 1 in 5 children with severe acute malnutrition were enroled in treatment, despite 79% of health care centres offering SAM treatment ($author1$ et al. 2019). This highlights that even major changes in treatment protocols may not lead to the desired changes in coverage in the absence of high‐quality treatment that is accessible to the target population.

To try and reduce barriers to access, the WHO issued new guidance in 2023 that community health workers (CHW) can be directly involved in providing acute malnutrition treatment. However, the recommendation was made based on evidence with very low certainty, and it was recommended that future studies should examine the effectiveness of CHW in managing wasting and/or nutritional oedema throughout the care pathway (Thompson et al. 2025; WHO 2015).

Integrated community case management (iCCM) is an existing healthcare programme which seeks to use CHW to diagnose and treat three common childhood illnesses: malaria, diarrhoea and acute respiratory infections (WHO/UNICEF 2012). iCCM has been shown to improve treatment coverage, although doubts remain about its impact on health outcomes and its cost‐effectiveness compared to facility‐based treatment (Collins et al. 2014; Daviaud et al. 2017; Oliphant et al. 2021). Nonetheless, there is a potential opportunity to build on this platform by adding the treatment of acute malnutrition, especially as there is mounting evidence that CHW can manage both moderate and severe uncomplicated acute malnutrition (Charle‐Cuellar et al. 2021; Alvarez Morán et al. 2018; López‐Ejeda et al. 2024; WHO 2023).

A 2024 trial in Kenya provided evidence that ICCM+ may be cost‐effective, though a recent Cochrane review underscored the need for further research on both the effectiveness and cost‐effectiveness of ICCM+ programmes (Ilboudo et al. 2024; Papadopoulou et al. 2023). We therefore implemented a trial to provide further evidence on the effectiveness and cost‐effectiveness of acute malnutrition treatment provided via CHW in the context of Somaliland. The trial took place following a design process and pilot study, during which the treatment protocols, patient registers, and diagnostic tools were developed and tested (Yuusuf et al. 2023).

Our primary research question was whether the integration of a simplified combined approach for treatment of acute malnutrition into an integrated community case management platform (iCCM +) would lead to greater access to effective acute malnutrition treatment compared to facility‐based treatment alone. Our secondary question was whether the treatment provided through the iCCM+ platform would result in better outcomes.

## 2 Methods

### 2.1 Study Hypothesis and Outcomes

The study hypothesis was that integration of a simplified, combined approach for the treatment of acute malnutrition into an integrated community case management platform (iCCM +) would lead to greater access to effective acute malnutrition treatment compared to facility‐based treatment alone. The primary outcomes were defined as treatment coverage for (UNICEF. WHO. World_Bank. 2025) moderate acute malnutrition (MAM) or (Haseeb and Ali 2026) severe acute malnutrition. Acute malnutrition was defined using mid‐upper arm circumference (MUAC) or oedema, and treatment was defined as being currently enroled in a village‐level or facility treatment programme, measured using study records on the day of the data collection.

The secondary outcomes for the trial were defined as:

i. Number of cases of acute malnutrition diagnosed by FHW; measured using health worker record books over the study period.
ii. Recovery (Number of cases successfully discharged by the FHW or health facility as cured, divided by total discharges multiplied by 100); measured by household follow‐up interviews.
iii. Default proportion (Number of defaulters divided by total discharges multiplied by 100); measured by household follow‐up interviews.
iv. Relapse proportion (proportion of cured children who become malnourished during the trial follow‐up period); measured by household follow‐up interviews.
v. Death rate (Number of beneficiaries who died whilst registered in programme, divided by total discharges multiplied by 100); measured by household follow‐up interviews.
vi. Average length of stay in treatment programme (days). Length of stay is the number of days elapsed between admission and discharge. Average length of stay = sum of individual length of stay (recovered beneficiaries) in days/number of recovered beneficiaries; measured by household follow‐up interviews.
vii. Average gain in MUAC (mm/week); measured by household follow‐up interviews.
viii. Prevalence of GAM at baseline and endline; measured during baseline and endline surveys. The potential impact of providing malnutrition treatment on the diagnosis and treatment of other conditions was monitored by recording the number of diagnoses and diagnostic rate made by each FHW for the different conditions.
ix. Number of diagnoses and diagnostic rate (diagnoses per 1,000 children/month) for each condition (ARI, malaria, diarrhoea, and acute malnutrition); measured using health worker record books over the study period.

In addition to the secondary indicators that are included in the list above and were registered with ISRCTN, we also measured non‐response. Non‐response was defined as not meeting discharge criteria after 8 visits (16 weeks) following the initial diagnosis.

### 2.2 Ethical Approvals and Trial Registration

The Ministry of Health Development of the Republic of Somaliland (reference: MOHD/DG:2/1048/2023) and the Research Ethics Committee of UCL (project ID: 4684/004) granted ethical approval. The study was registered on October 3, 2023, with ISRCTN (ISRCTN31437934). This study is reported as per the Consolidated Standards of Reporting Trials (CONSORT) guideline.

### 2.3 Study Design

A non‐blinded, prospective, longitudinal, cluster‐randomised, controlled trial, using 37 villages, served by a single FHW, as the units of randomisation.

### 2.4 Population and Setting

The study was conducted in rural villages in the Maroodijeex region of Somaliland, within the districts of Hargeisa, Gabiley, and Faraweyne. These areas consist mainly of dispersed rural settlements with limited infrastructure and long distances between villages and health facilities.

The population is predominantly ethnic Somali, with Islam as the main religion, and Somali (Maxaa Tiri dialect) as the primary language spoken. Most households rely on pastoralism, agro‐pastoralism, petty trade, and casual labour for their livelihoods. The region lies within a semi‐arid ecological zone characterised by low and variable rainfall and periodic droughts, which contribute to vulnerability to food insecurity and acute malnutrition.

Primary healthcare services are provided through NGO supported Mother and Child Health (MCH) clinics and CHW, known locally as family health workers (FHW). FHW deliver iCCM services, including treatment of common childhood illnesses, health promotion, and referral to health facilities. However, access to facility‐based services can be limited by distance, transportation costs, and geographic dispersion of households, making community‐based services particularly important.

### 2.5 Village Selection and Randomisation

A village (Somali: tuulo) may be comprised of a number of bootos or individual compounds, and extend over quite a large area. It is estimated that the average tuulo area ranges from 20 to 50 km. Forty tuulo were found to lie within the study area and had an active FHW who was employed by the MOHD and supported by Save the Children; lay > 3 km and < 15 km from an SC‐supported referral facility; and used a SC facility as the main referral site for the FHW. One of these tuulo has been utilised in a prototype study to develop the operating model and test the feasibility of data collection and was excluded from the study randomisation process.

Tuulos were randomly selected from the list of eligible tuulos using random numbers generated in Excel. Following this stage, the eligible tuulo were randomly allocated to control (iCCM) and iCCM+ intervention. Prior to the randomisation process, village‐level approval for the study was sought from local administrative authorities and community representatives in each village. Randomisation was done by listing all the villages in order of their catchment health facility and supervisor, then assigning each village a unique ID number. A random number, either 0 for control or 1 for intervention, was assigned to the first tuulo in the list by generating a random number using an Excel RANDBETWEEN function. The rest of the tuulos were then assigned to a study arm group by choosing alternating members from the list.

### 2.6 Intervention

In the intervention arm, a treatment package (iCCM + ) for uncomplicated acute malnutrition was provided, for children 6–59 months, by FHW at the village level. In Somaliland, this cadre of health workers is referred to as FHWs. The treatment consisted of the provision of a peanut‐ based RUTF (brand name PlumpyNut), to treat both severe (SAM) and MAM cases without complications. SAM cases received a take home ration of two (92 g, 500 kcal) sachets a day and MAM cases one sachet. Presumptive treatment with Amoxicillin (two doses over 24 h for children 6–59 mo.) and Albendazole (single dose for children 12–59 mo.) was provided to SAM cases while MAM cases received only Albendazole. The dose provided was age dependent. The FHW was also trained to give health and nutrition messages to the caregiver.

MAM was diagnosed using a MUAC < 12.5 to 11.5 cm, while SAM was diagnosed using a MUAC < 11.5 cm and/or bipedal oedema. MUAC tapes were specifically designed for the iCCM+ intervention and contained the standard diagnostic cut‐offs and colour bands for MAM and SAM, but with two additional colour bands to help identify children with very low MUAC measurements (10.5–11.5 cm, dark pink; 9–10.5 cm dark red; and < 9.0 cm, bright red) (Hickman and Nene 2024). The tapes were not printed with numerical values. Children with SAM were given a fixed ration of 2 RUTF sachets per day while children with MAM received 1 sachet per day. Once started, children remained on the one‐ or two‐sachet treatment schedule until discharge or default. Carers were asked to bring their children to the home of the FHW for follow‐up visits every 2 weeks until discharge. MUAC measurements were taken during each follow‐up visit to assess progress.

Children with oedema, a MUAC less than 9.0 cm, lack of appetite, danger signs, deterioration in MUAC category between visits, or failure to progress to the next MUAC colour band within 3 visits, were referred to the linked health facility for further investigation and treatment. Children were discharged as recovered when their MUAC exceeded 12.5 cm for two consecutive visits and marked as a default if they did not come for treatment for three consecutive visits. A child was considered a non‐responder if they had not recovered after nine visits. Patient data was recorded in separate registers for SAM and MAM that had been specifically designed for the iCCM+ intervention (Hickman and Nene 2024).

### 2.7 Control

The iCCM+ treatment package was compared to the pre‐existing standard of care (iCCM) in which acute malnutrition was diagnosed by FHW using a standard MUAC tape, but all cases were referred to a Mother and Child Health (MCH) clinic for treatment. The same level of active case finding by FHW conducting home visits was designed to happen in both study arms. The study villages lay within the catchment areas of one of four MCH clinics. At the clinics, MAM was diagnosed using a MUAC 11.5 cm to 12.4 cm, or a weight‐for‐height z‐score >=‐3 and <‐1.5 (rather than the more widely used <‐2 z‐score criteria). SAM was diagnosed using a MUAC < 11.5 cm, or a weight‐for‐height z‐score <‐3.0, or nutritional oedema. Health facility staff used standard, 3 colour band MUAC tapes. Children were admitted into an Outpatient Treatment Programme (OTP) if they had SAM, or a Targeted Supplementary Feeding Programme (TSFP) if they had MAM. Children were referred on to hospital for in‐patient treatment if considered severely ill.

Diagnosis and treatment of pneumonia and diarrhoea, and detection of danger signs, followed by referral to a health facility, was done for FHW in both study arms according to the current national (Somaliland) iCCM protocols (MoHD 2024). No testing or treatment for malaria was provided as the incidence was considered to be very low.

### 2.8 Study Components and Participants

**Figure 1:**
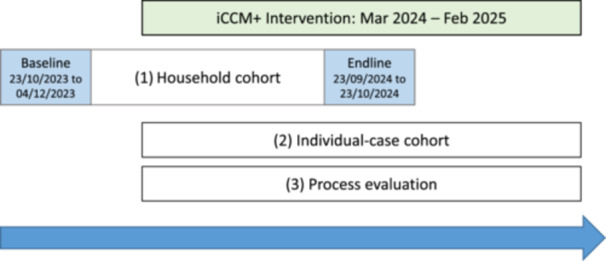
Study Schematic: Intervention and Data Collection Timeline.

The study consisted of three main components (Figure 1). To determine the primary trial outcome, treatment coverage, a closed household cohort was surveyed at baseline and endline. To determine treatment outcomes, an open cohort of individual cases of acute malnutrition, which had been identified by FHW in control and intervention villages, was followed up each month by the research team. A process evaluation was also conducted, and the main results will be reported separately.

### 2.9 Household Cohort

The unit of sampling was the household with a closed cohort of households followed from baseline through endline. The cohort was open to individuals entering or leaving the trial. The household cohort comprised all households within the selected villages that had consented to participate and had an eligible child at baseline. The number of households per village varied according to population size, varying from 17 to 131 households.

Only households with children aged *< *5 years were sampled. If a household was found not to have children aged *< *5 years, the household was skipped. We collected data on household characteristics, on children aged *< *5 years, and their mothers or primary carers. The data were gathered from household heads and mothers/primary carers and included questions on household demographics; language usage, morbidity; access to healthcare; water, sanitation, and hygiene, and vaccination status.

We also collected anthropometric data from children aged 6–59 months to describe their nutritional status and the prevalence of acute malnutrition, and assessed treatment coverage by asking if children with a MUAC < 12.5 cm and/or oedema, i.e. with global acute malnutrition (GAM), were currently enroled in an outpatient treatment programme (OTP) or TSFP. Children identified with a low MUAC and/or oedema were referred to nutrition centres for treatment.

### 2.10 Individual Case Cohort

Monthly visits were made by the research team to each village. On each visit, the team reviewed the FHW registry and extracted data from any new or updated records. The research team then located each case and conducted a home visit. Data was collected at the household level and included mother/carer reports of programme attendance, receipt of nutrition products (RUTF, RUSF or other SFP foods), and medication. MUAC, weight, height, and nutritional oedema measurements were also taken during household visits by the research team. Some cases were initially identified and reported by FHW but could not be found by the research team visits. There were 89 such cases in the intervention arm and 49 cases in the control arm. These were not included in the analysis. Ongoing case tracking was done throughout the duration of the 12‐month intervention period and captured data on all cases of acute malnutrition that were identified by the FHW. The data was collected during household visits between March 2024 and February 2025. We estimated each malnutrition case would need approximately 3 months to reach an outcome, so the last month of recruitment for newly identified acute malnutrition cases was November 2024.

The diagnostic accuracy of the FHW was assessed by comparing the MUAC values recorded during the monthly household visits by the research team and the MUAC category recorded by the FHW. To allow for the time delay between the FHW measurement and the household visit by the research team, the average population MUAC increase over that period was subtracted from the value recorded during the household visit. After this subtraction was done, the diagnosis of each case was reviewed to see if the FHW had correctly assigned the case to the normal, MAM or SAM categories.

### 2.11 Process Evaluation

A process evaluation was conducted to better understand the context in which the intervention was implemented, and document how the intervention was implemented compared to how it was planned. The main results from the process evaluation will be reported in a separate paper.

### 2.12 Research Team Training

To ensure the collection of high‐quality data in the household and child cohorts, a 2‐week training was implemented for enumerators and supervisors prior to the start of both data collection periods. The enumerators and supervisors were identified by the MOHD but hired by Save the Children to act exclusively as data collectors for the study. They were supported closely by UCL project staff, who provided a mix of online and face‐to‐face support during the field work, as well as designing and leading the training.

During the training, participants were trained on the causes and consequences of acute malnutrition, as well as the objectives and design of the study, along with instruction and practice on anthropometric data collection and use of digital data collection tools. We piloted the questionnaires and performed a standardisation test in a nearby health centre, before the baseline and endline data collection periods.

### 2.13 Field Procedures and Data Handling

For the household cohort, we collected data using a structured questionnaire, translated into the local Somali language, on mobile devices running the Android operating System and Open Data Kit. Data was uploaded by Wi‐Fi to servers run by ONA. Prior to analysis, data was downloaded from the severs as.csv files. All teams were supervised during field data collection, and a daily team brief was held online with study supervisors in Hargeisa and/or London. For the individual‐case cohort, we collected data using paper forms. These paper forms were digitally captured (photographed), and data entry was conducted by the data manager in the office.

### 2.14 Household Characteristics

To understand household composition, we collected data on the number of members and the number under 5 years of age, and whether the household was female‐headed. We also asked about phone ownership. The main sources of drinking water were asked and the frequency with which the household experiences inadequate drinking water. Lastly, we enquired about the defecation site used by the household, using locally available options as possible responses.

### 2.15 Women and Child Characteristics

We asked about the age and main language of the children's mothers, as well as the number of children under 5 years that she cared for. Questions relating to each child included sex and age, possession of a health record card, and their vaccination history. We collected data on vaccination coverage, based on the Somalia Expanded Programme on Immunisation (EPI) schedule by checking the child's health card at baseline and endline, or by others/caregivers recall if no health card was available or there was no entry for a particular vaccine. We also asked questions about children's health problems and morbidity, and where the carer consulted in the event they sought care.

### 2.16 Child Age and Anthropometry

We asked the caregivers if they knew the child's exact date of birth. Where that was not possible, we ascertained the children's month and year of birth using a calendar of local events. We measured, in duplicate, weight, length/height, and MUAC for children in the household cohort and MUAC for children in the individual‐case cohort, and obtained the mean. Weight was measured to 100‐g precision using an electronic scale (SECA model 870). Length and height in children aged *< *24 months and > 24 months, respectively, were measured to 1‐mm precision using a stadiometer (Infant/Child/Adult ShorrBoard). MUAC was measured on the left arm to 1‐mm precision using a TALC‐UK insertion tape. Presence of oedema in children in both cohorts was recorded if an imprint remained in both feet after pressing them with the thumbs for 3 s. Children's age in both cohorts was obtained using a calendar of events and was rounded to the nearest month. The anthropometric indices weight‐for‐length/height *z‐*score (WHZ) and height‐for‐age *z‐*score (HAZ) were calculated using the Stata *zanthro* command [34], and extreme values were flagged and excluded from analysis according to the cutoffs: mean WHZ/WLZ ± 4 SD.

### 2.17 Sample Size

The sample size was calculated using the Stata v17 command *clustersampsi*. In the initial calculation, we assumed there would be 7 cases of acute malnutrition (MAM and severe acute malnutrition combined) in each of 30 clusters, with a treatment coverage in the control arm of 50%. We used an ICC of 0.06 and a coefficient of variation for the size of the clusters of 0.72. The detectable effect size of the iCCM + intervention under those assumptions was 24% points, i.e. the study would be able to detect an increase in treatment coverage if it increased to 74% or greater. A 24% or better increase in treatment coverage would likely represent a programmatically significant improvement that would warrant scale‐up of the iCCM+ approach, assuming the economic analysis was also supportive. After baseline data collection commenced, it became apparent that the measured GAM prevalence was somewhat lower than assumed. After half the baseline survey had been completed, we estimated that the actual prevalence would be closer to 3%, and we therefore expected to find about three cases per cluster, rather than 7. Treatment coverage was estimated to be about 26% rather than the previous assumption of 50%. A decision was taken to increase the number of clusters from a total of 30 to 38. This resulted in a newly calculated detectable effect size of 26% points. One cluster had to be dropped following randomisation, leaving a total of 37 clusters in the combined study arms.

### 2.18 Data Analysis

We undertook data analysis using Stata v18. Prevalence estimates at baseline were computed accounting for the trial cluster design using the Stata complex survey svy commands, and differences in means and proportions were tested for using the lincom command. For variables collected at baseline and endline, we assessed impact using mixed effects, multilevel, logistic regression models. Models included cluster (village) and as random‐effects and dummy variables denoting the time point (baseline or endline) and intervention as fixed‐effects in the model. All models included baseline data to account for any observed differences at baseline and were adjusted for age and sex. The absolute difference in coverage (effect size) was calculated as the regression‐adjusted estimate using the *margins* command.

Analysis of case data was performed using the Stata complex survey svy commands and differences in means and proportions were tested for using the lincom command. To assess diagnostic accuracy, we compared the MUAC category recorded by the FHW in the register with the MUAC measurements taken by the research team during a subsequent household visit. As the household visit may have taken place some days or weeks after the diagnosis by the FHW a correction was applied to allow for any increase in MUAC since the diagnosis was done. This potential increase was estimated using data on the 250 cases for which we had more than 3 MUAC measures. A time series regression was calculated in Stata using the *xtreg* command and the regression coefficient was used to calculate the predicted MUAC measure at the time of diagnosis.

We conducted an intention to treat analysis. *P* values were considered significant at values < 0.05 and all confidence intervals are calculated at the 95% level.

## 3 Results

**Figure 2:**
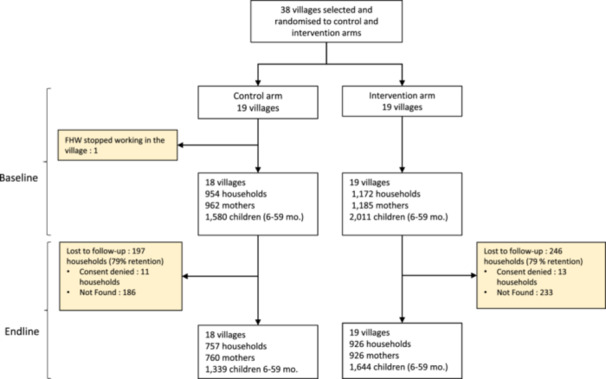
Trial flow chart.

The study flow chart is shown in Figure 2. Thirty‐eight villages were selected and randomised with 19 villages in each arm. Following randomisation, the FHW stopped working in one village and could not be replaced. This village was therefore dropped before baseline data collection took place. Overall enrolment and participation in the study was high at baseline with a total of 2126 households being enroled and surveyed, and no refusals. At endline, 11 households refused consent in the control arm and 13 in the intervention arm. However, a comparatively large number of households could not be found, resulting in an overall loss‐to‐follow‐up of 21% in the combined arms.

The baseline characteristics of the trial participants are shown in Table 1 and were generally well balanced between arms. Overall, the average household size was 7.3 persons with 1.8 children under 5 years. Sixty‐three percent reported having a female head of household and 87% of households owned a phone. The high level of mobile phone ownership allowed for its use by the research teams in locating household members and following up cases. The main source of drinking water for the majority of households was a berkad, a type of manmade pond, commonly used for water storage in Somaliland, with wells or springs used by about 14% of households. About 80% of households reported never experiencing an inadequate water supply. However, open defecation was widely practiced with 8 out of 10 households reporting that the field was their main defection site.

**Table 1:** Baseline characteristics.

Characteristics	Control		Intervention		Combined	
	Mean/%	95% CI	Mean/%	95% CI	Mean/%	95% CI
(a) Households						
Number of villages (*n*)	18		19		37	
Number of households (*n*)	954		1172		2126	
Households per village	55.9	48.0; 63.9	81.9	62.5; 101.2	70.2	57.0; 83.5
Household size	7.2	6.8; 7.6	7.3	7.1; 7.5	7.3	7.1; 7.4
Female head of household (%)	64.2	46.3; 78.8	62.0	43.7; 77.5	63.0	50.1; 74.2
Children aged < 5 in the household	1.8	1.7; 1.9	1.9	1.8; 1.9	1.8	1.8; 1.9
Phone ownership (%)	87.9	82.6; 91.8	86.3	82.8; 89.3	87.1	84.1; 89.6
(b) Mothers						
*n*	962		1,185		2,147	
Age (years)	31.1	30.2, 31.9	32.5	31.5, 33.5	31.9	31.1, 32.6
Number of children < 5 per woman	1.7	1.7, 1.8	1.8	1.7, 1.9	1.8	1.7, 1.8
Mother's main language						
Maay (%)	0.1	0.0, 0.7	0.2	0.0, 1.1	0.1	0.0, 0.6
Mahatiri (%)	99.9	99.3, 100.0	100.0	—	100.0	99.6, 100.0
Jido (%)	0.0	—	0.0	—	0.0	—
Garre (%)	0.0	—	0.0	—	0.0	0.0
Dabare (%)	0.0	—	0.0	—	0.0	—
Mushunguli (%)	0.1	0.0, 0.8	0.0	—	0.0	0.0, 0.4
Other (%)	0.3	0.1, 1.0	0.1	0.0, 0.7	0.2	0.1, 0.5
(c) Children 6–59 months (*n*)						
*n*	1580		2,011		3,591	
Sex (% female)	47.4	44.3, 50.5	50.2	47.7, 52.6	49.0	47.0, 51.0
DOB known (%)	8.5	4.8, 14.8	5.7	3.1, 10.2	7.0	4.6, 10.5
Mean age (months)	32.3	31.3, 33.2	33.3	32.7, 34.0	32.9	32.3, 33.5
Proportion of children with a health record card (%)	11.1	7.4, 16.5	13.0	7.9, 20.6	12.2	8.7, 16.8
Proportion of children with any vaccinations (%)	73.8	61.7, 83.1	77.6	65.2, 86.5	75.9	67.4, 82.7
Carer is aware of the FHW in the village (%)	95.6	91.3, 97.9	90.9	74.1, 97.2	93.0	84.0, 97.1
Carer knows where the FHW lives (%)	94.9	90.6, 97.3	90.5	74.2, 96.9	92.4	83.8, 96.6
Proportion of children with any health or nutrition problem in the last 4 weeks (%)	10.5	5.9, 17.9	12.7	7.4, 20.8	11.7	7.9, 17.0

The characteristics of the mothers/carers of children aged 6‐59 mo. are shown in Table 1b. The average age was 32 years with each woman having an average of 1.8 children under 5 years old. Almost all women spoke the main Somali language, Maxaa Tiri, with a few reporting Maay, Mushunguli, or other languages or dialects as their main language.

The characteristics of children between 6 and 59 months are shown in Table 1c. The mean age was 33 months, and the sex distribution was not significantly different from 1:1. Only 12% of children had a health record card although 76% had received at least one vaccination. Awareness of the village FHW and knowledge about where she lived was high at over 90%. Amongst children that had had a health problem within the last 4 weeks, 65% had consulted the FHW, with 26% going to an MCH clinic and 9% using a pharmacy. However, for those who had not sought health care the main reasons given were the service was too far away. The full characteristics of households, women, and children are provided in Web Table 1.

The nutritional status of children over the course of the study is presented in Table 2 with the prevalence of acute malnutrition shown for low MUAC and/or low eight‐for‐height combined. Full anthropometric results are provided in Web Table 2. Very few cases of nutritional oedema were observed and mean weight, height, and MUAC were well balanced between arms at both baseline and endline.

**Table 2:** Child nutritional status by arm at baseline and endline in the household cohort.

	Baseline						Endline					
Characteristic	Control		Intervention		Combined		Control		Intervention		Combined	
	mean/%	95% CI	mean/%	95% CI	mean/%	95% CI	mean/%	95% CI	mean/%	95% CI	mean/%	95% CI
*n*	1580		2011		3591		1341		1642		2983	
Children with bilateral, pitting oedema (%)	0.2	0.1, 0.6	0.1	0.0, 0.4	0.1	0.1, 0.3	0.0	—	0.1	0.0, 0.4	0.0	0.0, 0.2
MUAC (cm)	14.9	14.8, 15.0	14.8	14.5, 15.0	14.8	14.7, 15.0	15.2	15.0, 15.3	15.0	14.8, 15.2	15.1	14.9, 15.2
Height (cm)	89.0	88.3, 89.7	89.8	89.3, 90.3	89.5	89.0, 89.9	90.2	89.5, 90.9	90.2	89.4, 90.3	90.2	89.7, 90.7
Weight (kg)^a^	11.9	11.7, 12.1	12.1	11.9, 12.2	12.0	11.9, 12.1	12.2	12.0, 12.3	12.1	11.9, 12.3	12.1	12.0, 12.2
*Combined acute malnutrition prevalence by weight‐for‐height or MUAC, and/or oedema*												
Moderately acutely malnourished % (*n*)	10.8	9.1, 12.9	11.2	9.0, 13.8	11.0	9.5, 12.7	11.1	8.9, 13.7	10.2	8.8, 11.7	10.6	9.3, 12.0
	(171/1580)		(225/2011)		(396/3591)		(148/1,41)		(167/1642)		(315/2,983)	
Severely acutely malnourished % (*n*)	3.5	2.9, 4.2	2.9	2.3, 3.6	3.1	2.7, 3.6	2.3	1.7, 3.1	2.8	1.9, 4.0	2.6	2.0, 3.3
	(55/1580)		(58/2011)		(113/3591)		(31/1341)		(46/1642)		(77/2983)	
Global acute malnutrition % (*n*)	14.3	12.5, 16.3	14.1	11.7, 16.9	14.2	12.6, 15.9	13.4	11.0, 16.2	13.0	11.2, 15.0	13.1	11.6, 14.8
	(226/1580)		(283/2011)		(509/3591)		(179/1341)		(213/1642)		(392/2983)	

^a^ 150 g was subtracted from the measured weight to account for clothing.

Prevalence of acute malnutrition by MUAC and/or oedema was low, falling from about 3% at baseline to 1% at endline. However, the prevalence of acute malnutrition by weight‐for‐height and/or oedema was moderately high at 13% and 12% at baseline and endline, respectively. According to WHO criteria the population was experiencing serious or high levels of acute malnutrition (de Onis et al. 2019). In the baseline survey, the ICC for GAM by MUAC was 0.0149 and the ICC for GAM by WHZ was 0.0031.

This fourfold difference was greater than expected and implied that treatment of acute malnutrition diagnosed using MUAC and/or oedema could only be expected to achieve a maximum of approximately 20% coverage of the total burden of acute malnutrition in this population.

The combined prevalence of acute malnutrition by weight‐for‐height or MUAC and/or oedema is also shown in the table. The combined prevalence was only slightly higher than the prevalence determined using only WHZ. The children identified using either measure were, in principle, eligible for treatment at health facilities within the study area, where diagnosis was performed using both weight‐for‐height and MUAC, as well as nutrition oedema.

The analysis of treatment coverage based on diagnosis of MAM and SAM using MUAC was the main outcome of the trial, and is shown in Table 3. The regression model shows that coverage for MAM increased in both study arms, with an adjusted change of 16% points in the intervention arm, but there was no significant difference between arms. Analysis of the change in treatment coverage for SAM was not performed as there were so few cases of SAM detected by the FHW. The combined treatment coverage for MAM and SAM was also analysed but again, there was no significant difference between arms, despite an adjusted difference of 20 percentage points. A retrospective power calculation using the *clustersampsi* function in Stata v18 showed that given the small prevalence of GAM by MUAC, the study would only have been capable of detecting a difference in treatment coverage of 42% or greater.

**Table 3:** Main outcomes—treatment coverage for all cases identified in the household cohort.

	Baseline				Endline				Intervention effect			
	Control		Intervention		Control		Intervention					Adjusted difference (% points)
	% (*n*)	95% CI	% (*n*)	95% CI	% (*n*)	95% CI	% (*n*)	95% CI	Odds ratio	95% CI	*p*‐value	
*Nutrition programme enrolment for children diagnosed using MUAC and/or oedema*												
SFP % (*n*)	15.2	3.9, 44.3	6.5	1.0, 31.8	7.7	0.8, 45.2	21.1	5.9, 53.2				
Global acute malnutrition	27.3	10.7, 54.0	22.6	14.1, 34.1	30.8	12.1, 59.0	47.4	27.6, 68.0	2.55	0.37, 17.81	0.344	19.6
	(9/33)		(14/62)		(4/13)		(9/19)					
Moderate acute malnutrition	26.9	8.5, 59.4	23.2	14.2, 35.5	30.0	10.1, 61.9	47.1	27.7, 67.4	2.23	0.22, 22.14	0.494	16.1
	(7/26)		(13/56)		(3/10)		(8/17)					
Severe acute malnutrition	28.6	5.8, 72.0	16.7	1.1, 79.0	33.3	1.1, 95.7	50.0	1.2, 98.8	—	—	—	—
	(2/7)		(1/6)		(1/3)		(1/2)					

*Note:* Analysis was conducted using multi‐level mixed effects logistic regression, adjusted for age and sex as fixed effects and village as a random effect. Adjusted difference was calculated using the Stata margins command. This calculation was omitted for severe acute malnutriiton due to the very small sample size.

The analysis of treatment outcomes, the secondary outcomes of the trial, is presented in Table 4. During the follow‐up period of the study (Figure 1), 253 cases of GAM by MUAC were identified by the FHW. Enrolment for treatment after identification was significantly better in the intervention villages. All of the identified cases were enroled for treatment in these villages but only 28% of cases in the control villages attended health facilities for treatment. However, the proportion of cases that enroled for treatment and then successfully recovered was similar between the arms, as was the proportion that defaulted. For cases that recovered, the length of stay appeared to be shorter in the intervention villages (46 days vs 67 days) but there was no significant difference between arms. We note that as outcome was unknown for 40% of the cases that started treatment in the control arm versus 7% in the intervention arm comparison of treatment outcomes between the two arms should only be made with caution.

**Table 4:** Secondary outcomes—treatment outcomes for all cases identified by family health workers (*n* = 253).

Outcome	Control			Intervention			Difference		
							(intervention ‐ control)		
	*n*	value	95% CI	*n*	value	95% CI	Value	95% CI	p‐value
Cases identified by FHW	89			164					
Referral to MCH clinic	89/89	100.0%	—	11/164	6.7%	2.1, 19.4			
Started treatment	25/89	28.1%	16.5, 43.5	164/164	100.0%	—	71.9%	58.1, 85.7	**< 0.001**
Referral onwards from MCH	2/25	8.0%	1.6, 31.9	0/164	0.0%	—			
Outcome unknown after starting treatment	10/25	40.0%	17.7, 67.4	12/164	7.3%	2.5, 19.6			
Discharges	15/23	65.2%	34.4, 87.0	152/164	92.7%	80.6, 97.5	27.5%	2.4, 57.3	0.070
Recovery	10/15	66.7%	45.1, 83.0	94/152	61.8%	49.1, 73.1	4.8%	−28.1, 18.4	0.673
Non‐response	0/15	0.0%	—	8/152	5.3%	2.0, 13.2	5.3%	0.25, 10.28	**0.041**
Default	5/15	33.3%	17.0, 54.9	50/152	32.9%	22.2, 45.8	−0.4%	−23.6, 22.7	0.969
Relapse	0/10	0.0%	—	17/94	18.1%	9.2, 32.5	18.1%	6.5, 29.6	**0.004**
Deaths among those with known outcomes	0/79	0.0%	—	0/152	0.0%	—	—	—	—
Mean length of stay for recovered children (days)	10	66.6	40.7, 92.5	94	46.7	41.9, 51.5	−19.9	46.3, 6.48	0.132
Mean MUAC gain (mm/week) in discharged children*	15	0.28	0.02, 0.54	152	0.30	0.21, 0.40	0.03	−0.12, 0.18	0.634
Misdiagnosis	60/89	67.4%	56.7, 76.6	127/164	77.4%	65.3, 86.2	10.0%	4.5, 24.5	0.170

*Note:* Bold values indicate statistically significant differences.

* 4 Children with < 2 recorded MUAC measures were excluded in the calculation of MUAC velocity.

No cases of relapse (re‐admission) were seen in the control arm but 18% were recorded in the intervention arm. No deaths were reported in either study arm. The MUAC gain velocity was 0.3 mm/week with no detectable difference between study arms.

The diagnostic accuracy of the FHW was assessed by monthly household visits by the research team, during which the identified cases were re‐measured and the MUAC value compared against the value recorded by the FHW. This comparison revealed that a high proportion of cases had been misdiagnosed by the FHW with more than 79% of cases being diagnosed as GAM while they were very likely to have had a MUAC > = 12.5 cm. In the control arm, the level of misdiagnosis was 67%. The treatment outcomes after exclusion of the misdiagnosed cases are shown in web Table 3. Probable reasons for this low diagnostic accuracy were explored using qualitative research methods, and the results will be reported elsewhere (in preparation).

The total number of cases of acute malnutrition in children (aged 6–59 mo.) from the study villages that were treated at the 4 MCH clinics during the study period was determined by a review of their clinic treatment registers. This revealed that 352 additional cases were treated for malnutrition without being recorded in the FHW registers, suggesting that they bypassed the FHW and sought health care directly at the clinic (Web Table 2). 345 (98%) of these cases met the local criteria for programme admission based on a WHZ diagnosis (WHZ < −1.5), while 78 (22%) met the criteria for admission based on MUAC < 12.5 cm. Less than 2% of cases were admitted on the basis of MUAC without also meeting the WHZ criteria.

These results indicate that during the study period FHW referral or treatment resulted in only 253/606 (42%) of the malnutrition treatments that were delivered to children in the study area.

## 4 Discussion

### 4.1 Key Results

This study is one of only a few that have formally trialled the iCCM+ approach for treating acute malnutrition in young children. We found evidence that treatment uptake is increased by making treatment available at village‐level. While there was a trend towards increased treatment coverage, there was no statistically significant difference between study arms. This may have been because the study was underpowered due to the unexpectedly low numbers of acutely malnourished children that were detected using MUAC‐based diagnostic criteria. The large discordance between MUAC and WHZ‐based diagnosis in this population raises questions about the efficacy and equity of programmes that only utilise MUAC‐based diagnosis in the community, while both criteria are used at facilities. However, when assessing these issues, it should be noted that neither diagnostic approach is based on ‘gold standard’, outcome‐based criteria and the mortality risk for individual children depends on a range of factors (Ahmed et al. 2025). A unique feature of this trial was that cases of malnutrition, that has been identified by FHW were followed up at home by a separate research team and remeasured. This allowed for cross‐checking of the diagnostic accuracy of CHW and revealed serious concerns about misdiagnosis and overprescription of RUTF.

### 4.2 Comparison With Other Studies

While one of the major potential benefits of using CHW is to improve treatment coverage by making treatment more accessible and reducing the opportunity costs to carers, few studies had examined this assumption. Evidence from Mali and Mauritania indicates that treatment coverage for severe acute malnutrition can be increased by the introduction of CHW treatment, but that this effect is not seen in all locations and other factors may moderate its impact (Charle‐Cuéllar et al. 2021, 2022). In this study, we found a non‐significant trend towards increased treatment coverage for combined moderate and severe acute malnutrition.

A 2023 review found that identification and treatment of uncomplicated severe wasting in children by CHW may lead to similar or slightly poorer outcomes when compared to treatment by health professionals at facilities (Papadopoulou et al. 2023). In this study, we found that a higher proportion of diagnosed patients started treatment in the intervention arm, but the small sample size precluded any conclusions about the outcomes that were achieved.

Economic analysis has also indicated that decentralised treatment of acute malnutrition can be cost‐effective (Ilboudo et al. 2024; Cichon et al. 2025b). Further analysis will be published on the comparative marginal treatment costs that were documented in this current trial, where malnutrition treatment was integrated into a pre‐existing iCCM programme.

### 4.3 Strengths

The study design was robust, with a reasonable number of clusters per arm and a good sample size in terms of households and children. The study benefitted from a previously established partnership between an operational NGO and an academic partner. The research teams were well‐trained, and their time was dedicated to the research project. Although field supervision was challenging, due to long travel times between villages and health centres and the poor road conditions, teams were supervised on the ground and remotely by the UCL study team. There was a good level of involvement and engagement with the Somaliland Ministry of Health Development which helped ensure the relevance of the study to national priorities and an appropriate design and implementation approach.

### 4.4 Limitations

A major limitation of the study was that the achieved sample size meant that it was underpowered. When initially calculating the required sample size data were only available from surveys that had used WHZ to measure the prevalence of acute malnutrition. MUAC and WHZ are two, largely, independent measures of acute malnutrition in young children and are known to produce very widely varying estimates of prevalence, with the use of MUAC typically producing a lower estimate (Leidman et al. 2019). However, it is relatively unusual for the ratio of MUAC: WHZ‐based prevalence estimates to be as low as was found in this population sample. This resulted in a much lower number of acute malnutrition cases being detected in the study villages using MUAC than had been expected. As described in the methods, following the preliminary analysis of the first half of the baseline survey was completed it became apparent that the GAM by MUAC prevalence was less than half what had been assumed. The number of clusters was increased up to the maximum feasible number, giving a detectable effect size of 26%. However, in the event, the increase in coverage in the intervention arm was only 19.6%, and no statistically significant difference was detected.

A closed household cohort was utilised to assess the change in treatment coverage. This meant that the sample may not have been representative of the total population over the course of the 6 months intervention period, for example, new households arriving in the study area would not have been included in the endline assessment of coverage. This approach had both advantages and disadvantages. One disadvantage was that the endline assessment of coverage would not have included new households, even if members of the household had been included in the individual case cohort.

### 4.5 Interpretation

#### 4.5.1 Underdiagnosis Due to MUAC Only Approach

As described above, we observed a much higher prevalence when using WHZ rather than MUAC to determine anthropometric status. Large differences are not unusual, and the relationship between these two nutritional indicators has been described in a number of papers (Leidman et al. 2019; Grellety and Golden 2016). However, the four‐fold variation seen in this context is on the higher end of documented differences and resulted in a high level of undertreatment of the total acute malnutrition cases at the village level, where diagnosis was done using only MUAC and/or oedema. At health facilities, admission criteria included both MUAC and WHZ measurements, so some children who would not qualify for treatment at the village level may have been able to access care at the facility level. This possibility is supported by the finding from clinic records that they treated an additional 352 cases that had not been recorded by the FHW at the village level (web Table 2). However, it should be borne in mind that the MCH clinics were using a WHZ admission criteria of < −1.5 z‐scores. This contrasts with the standard recommended threshold of < −2.0 z‐scores and resulted in the admission of 29% more cases than would have been seen if standard diagnostic thresholds had been applied.

#### 4.5.2 Overdiagnosis Due to Misdiagnosis by FHW

This is one of the very few studies to have used a research team to remeasure children who were diagnosed in an iCCM+ programme. The very high levels of probable misdiagnosis that were detected points towards the provision of inadequate training and supervision for the FHW. It also raises questions about the accuracy of diagnosis and the possibility of overtreatment occurring in other, similar programmes in low‐resource settings. However, it is important to note that no remeasurement of cases were performed on children who were diagnosed at facilities. Therefore, a comparison of diagnostic accuracy between FHW and clinic‐based health workers was not possible. While the issue of misdiagnosis was identified during an interim analysis of trial data, the health risks involved in treating children who failed to meet the MUAC < 12.5 cm criteria (false positives) were considered very low, and the trial was not stopped. It was not possible to know from the available data if measurement error may have also led to misdiagnosis of true positive cases. However, observations of measurement technique and interviews with FHW strongly suggested that the possibility of false negatives was very low.

### 4.6 Generalisability

While some of the issues identified during this trial may be specific to the context of Somaliland, the trial was conducted in a country which has many of the characteristics associated with populations experiencing malnutrition, including an under‐resourced healthcare system, the use of CHW, the use of WHO protocols on the management of acute malnutrition, and service provision by multiple INGOs. Moreover, the issues of misdiagnosis, coexistence of different diagnostic and treatment approaches within the health system, and the challenges of ensuring effective training and supervision, pointed out by the study, may well be taking place in other settings. For these reasons, the findings of this study are likely to be of relevance and should be considered by other countries in the region and more widely.

WHO guidelines made a conditional recommendation in 2023 that acute child malnutrition can be assessed, classified, and managed by CHW (WHO 2023). The attached conditions were that the CHW received adequate training and regular supervision of their work. The results from this trial suggest that the challenges of ensuring that these elements are adequately implemented in a large and highly dispersed population setting are substantial.

## 5 Conclusions

The regression‐adjusted treatment coverage was 19.6 percentage points higher in the iCCM+ intervention arm, but the difference was not statistically significant. A trial with a larger sample size is therefore recommended in this context.

Within the study area, the majority of malnutrition cases were diagnosed and treated at the facility level, without the involvement of FHW. There was a high level of probable misdiagnosis by FHW, suggesting inadequate training and supervision.

## Author Contributions

**Gael Cronin:** conducted data analysis, supervised data collection, and co‐wrote the manuscript. **Mohamed Jelle:** managed data collection, and contributed to study design and paper writing. **Shukri Ali:** execution of study/acquisition of data. **Yuusuf Siciid:** execution of study/acquisition of data. **Yayha Abdillahi:** execution of study/acquisition of data. **Meftuh Omer:** conceptualisation, review and editing. **Adan Yusuf Mahdi:** conceptualisation, review and editing. **Saed Abdi Ibrahim:** conceptualisation, study execution. **Lilly Scholfield:** conceptualisation, study design, interpretation of results. **Emily Keane:** conceptualisation, interpretation of results. **Khalid Ali Ahmed:** conceptualisation, review and editing. **Andrew Seal:** led study design, contributed to data analysis, and co‐wrote the manuscript.

## Conflicts of Interest

The authors declare no conflicts of interest.

## Supporting information


**Table 1:** Characteristics of Participant Households and Children.
**Table 2:** Anthropometric Status of Trial Participants.
**Table 3:** Secondary Outcomes‐Treatment Outcomes for Cases Confirmed by Research Team (n = 66).
**Table 4:** Additional Cases of Acute Malnutrition Treated at MCH Clinics Serving the Study Villages.

## Data Availability

The data that support the findings of this study are openly available in UCL at https://rdr.ucl.ac.uk/.
