# Supplementary material for: Combining Treatment of Acute Malnutrition With Integrated Community Case Management: A Cluster‐Randomised Controlled Trial (SETiPlus)
Source: Matern Child Nutr. 2026 May 24;22(3):e70203. doi: 10.1111/mcn.70203 (PMC13205731; doi:10.1111/mcn.70203)

**Web Table 1: Characteristics of Participant Households and Children**

**Web Table 2: Anthropometric Status of Trial Participants**

**Web Table 3: Secondary Outcomes – Treatment Outcomes for Cases Confirmed by Research Team (n=66)**


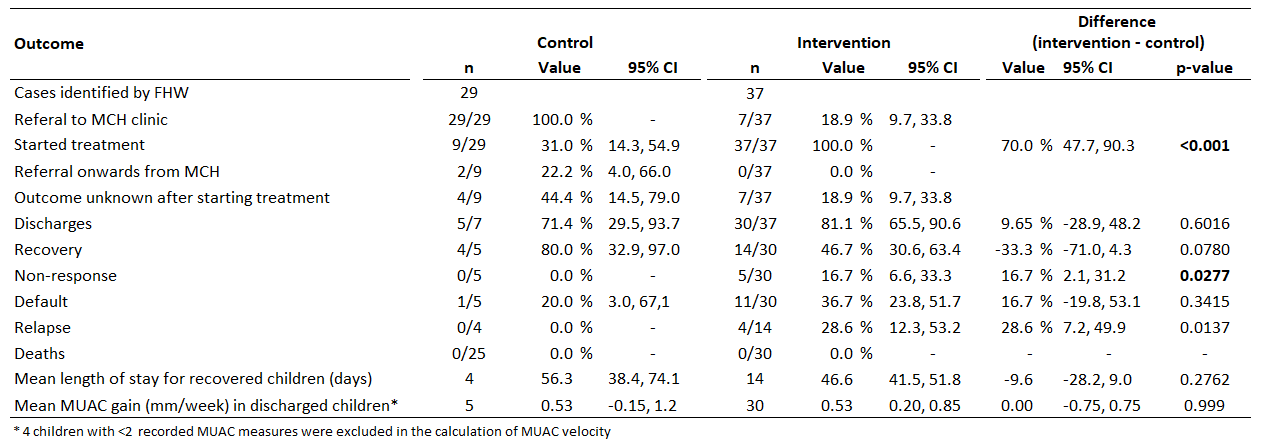


**Web Table 4: Additional Cases of Acute Malnutrition Treated at MCH Clinics Serving the Study Villages**


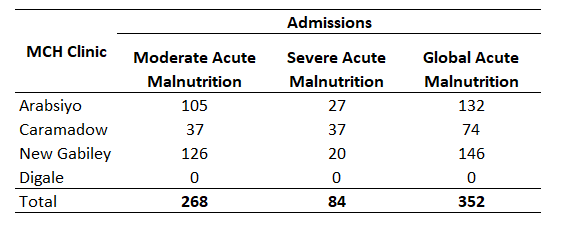

Supplement: Supplementary file 1 — Table 1: Characteristics of Participant Households and Children. Table 2: Anthropometric Status of Trial Participants. Table 3: Secondary Outcomes‐Treatment Outcomes for Cases Confirmed by Research Team (n = 66). Table 4: Additional Cases of Acute Malnutrition Treated at MCH Clinics Serving the Study Villages. [file MCN-22-e70203-s001.docx]
